# ^1^H NMR is not a proof of hydrogen bonds in transition metal complexes

**DOI:** 10.1038/s41467-019-09625-9

**Published:** 2019-04-09

**Authors:** J. Vícha, C. Foroutan-Nejad, M. Straka

**Affiliations:** 1 Centre of Polymer Systems, University Institute, Tomas Bata University in Zlín, Třída T. Bati, 5678, CZ-76001 Zlín, Czech Republic; 2 Department of Chemistry, Faculty of Science, Masaryk University, Kamenice 5, CZ–62500 Brno, Czech Republic; 3 Institute of Organic Chemistry and Biochemistry of the Czech Academy of Sciences, Flemingovo nám. 2, CZ-16610 Prague, Czech Republic

**Arising from** M. A. Bakar et al. *Nature Commun.* 10.1038/s41467-017-00720-3 (2017)

Hydrogen bonding to gold(I) and its effect on the structure and dynamics of molecules have been a matter of long debate.^1^ A number of X-ray studies have reported gold compounds with short Au^I^···H contacts, but solid spectroscopic evidence for Au^I^···H bonding has been missing.^1^ Notably, during the revision of this work, Bourissou et al.^2^ and Straka et al.^3^ have provided evidence of true intramolecular Au^I^···H hydrogen bonds in [Cl–Au–L]^+^ complexes, where L is a protonated N-heterocyclic carbene. The studied compounds feature intramolecular Au^I^···H^+^–N bonds detected by means of NMR^2^ and infrared spectroscopies.^2,3^

Previously in this Journal, Bakar et al.^4^ reported compound **1** (Fig. 1a) with four short Au···H contacts (2.61–2.66 Å X-ray determined). Assuming the central cluster in **1** to be [Au_6_]^2+^ and observing the ^1^H (^13^C) NMR resonances at respective H(C) nuclei in **1** highly deshielded with respect to precursor **2** (Fig. 1b), the authors concluded that “the present Au···H–C interaction is a kind of hydrogen bond”, where the [Au_6_]^2+^ serves as an acceptor”.

**Fig. 1:**
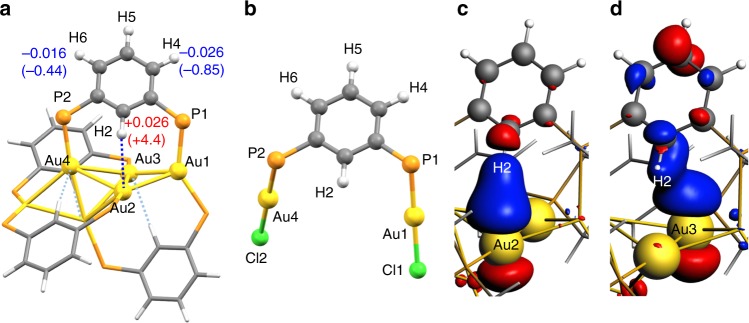
Schematic structures of **1** and **2**, and selected ETS-NOCV channels in **1**. **a** The schematic structure of **1** with indicated differences in calculated charges and experimental ^1^H NMR shifts^4^ (in brackets) between **1** and **2** for selected hydrogens. **b** Precursor **2**. ETS-NOCV channels in **1′** corresponding to **c** Au2···H2–C2 interaction, and **d** side-on Au3···H2–C2 interaction. Large P(Ph)_2_ sidechains are omitted for clarity. Cutoff of 0.0002 is used in **c** and **d**

Here, we show that the Au_6_ cluster in **1** bears negative charge and the Au···H contacts lead to only a rather weak (~1 kcal mol^−1^) auride-like···hydrogen bonding interaction. In addition, computational analysis of NMR chemical shifts reveals that the deshielding effects at respective hydrogen nuclei are not directly related to Au···H–C hydrogen bonding in **1**. It is well known that interactions of hydrogen with transition metals compounds may influence the ^1^H NMR shifts in unexpected ways.^5^

In the following, we analyze Au2···H2–C2 contact in **1**, which is one of the four Au···H–C contacts in the molecule (Fig. 1a). Computational methodology is described in Supplementary Information. The calculated C–H distances (1.08 Å) in **1** are about 0.15 Å longer than those derived from the X-ray structure (0.95 Å) as proposed in ref. ^4^. The calculated minimum Au···H distances (2.61–2.62 Å) are in excellent agreement with the reported ones (2.61–2.65 Å). To afford computational analysis of NMR chemical shifts^7–9^ (δ), the P(Ph)_2_ groups in **1** and **2** were replaced by P(CH_3_)_2_ groups in model systems **1′** and **2′** (Fig. 1). Such changes are known to have minimum impact on δ(^1^H).^8^ Notably, the absence of the bulky P(Ph)_2_ ligands causes rotation of the central phenyl groups away from the Au_6_ cluster. The Au···H distances increase from 2.6 Å to 2.77 Å and the Au–H2–C2 angles bend from 167° to 144° (Supplementary Fig. 1). The short Au···H–C contacts in **1** are thus likely enforced indirectly by sterically demanding P(Ph)_2_ ligands that potentially stabilize the whole cluster via dispersion interactions among themselves.^10^ To avoid these undesirable changes in calculations, we fixed the core of **1** and **2** in optimization of **1′** and **2′** and only methyl groups were optimized.

Quantum theory of atoms in molecules (QTAIM) analysis of **1′** shows a low ED (0.016 e.bohr^−3^) with positive Laplacian (0.037 e.bohr^−5^) at the line critical point (LCP) of Au2···H2 interaction. These values are less than a half of those for reported Au···H^+^–N bonds.^2,3,6^ Small electron exchange between Au2 and H2 of 0.07 e (e = electron) is consistent with a dispersive interaction.^11,12^ The direction of the charge transfer in Au2···H2 interaction is from Au2 to H2 (0.04 e, Supplementary Table 2) similar to auride···hydrogen weak interaction.^1^ All four Au atoms in contact with phenylene H2 atoms (Au2–Au5) in **1′** have negative charge, about −0.15 e (using QTAIM, Supplementary Table 3). Thus, the Au_6_ cluster, although formally a di-cation, strongly pulls the electron density from the ligands in **1**.

Extended transition state-natural orbitals for chemical valence (ETS-NOCV)^13^ analysis of **1′** reveals a weak Au2···H2–C2 interaction channel (0.9 kcal mol^–1^, Fig. 1c), which is about 10 times less than for recently reported Au···H^+^–N bonds.^2,3^ Smaller, weakly stabilizing “side-on” interaction (0.4 kcal mol^–1^) is found between H2–C2 and Au3 6p orbitals (Fig. 1d). Notably, the Au2···H2–C2 channel (Fig. 1c) is also found in the fully relaxed structure of **1′** (0.8 kcal mol^–1^). This further points to a minimal stabilization effect of Au···H–C bonding in **1**.

The calculated differences in ^1^H chemical shifts between **1** and **2** (**1′** and **2′**) are in excellent agreement with the experimental ones, for H2 Δ_**1**–**2**_ = 4.4 ppm, Δ_**1′**−2**′**_ = 3.6 ppm, and Δexp_**1–2**_ = 4.4 ppm (absolute values are shown in Supplementary Table 1). The analysis of the NMR chemical shifts affordable only for **1’** and **2’** reveals that 1.6 of 3.6 ppm of calculated Δ_**1′**-2**′**_(H2) arises from the diamagnetic part of the NMR chemical shift, Δδ^dia^, which can be rationalized only by a depletion of electron density (ED) at the H2 nuclei. Molecular orbital (MO) analysis of Δδ^dia^ identifies that main part Δδ^dia^ (1.3 of 1.6 ppm) originates from four Au–P π-back-bonding MOs (Supplementary Fig. 2). No through-space interactions between Au2 and H2 can be seen in these MOs. Quite to the contrary, the HOMO and HOMO-1 have Au2–H2 antibonding character (Supplementary Fig. 2). Despite the fact that Au2 shares some ED with H2 (see above), the overall ED at the H2 decreases by 0.010 e bohr^−3^ from **2′** to **1′**. Notably, ED increases at H4 and H6. Changes in ED are reflected in NMR chemical shifts, as deshielding is observed at H2 and shielding is observed at H4 and H6. This interpretation is supported by the calculated differences of atomic charges (Supplementary Tables 3, 4), which correlate well with the reported experimental differences of δ(^1^H) at H2, H4, and H6 nuclei^4^ given in brackets in Fig. 1a.

The paramagnetic part of the Δ_**1′**−**2′**_ deshielding difference at H2 nuclei (2 ppm) is dominated by local Ramsey-type paramagnetic couplings^9,14,15^ between H2–C2 σ-bond (HOMO-1 of **1′** in Fig. 2) and vacant MOs* formed by π* C2 2p_y_ orbitals (e.g., LUMO + 5 of **1′** in Fig. 2). Mixing of Au3 6p* and 5d_z2_* atomic orbitals (AOs) with C2 2p_y_* in **1′**, which is not possible in **2′**, increases the MO ↔ MO* overlap in orbital magnetic couplings.^9,15,16^ This leads to the ~0.5 ppm larger paramagnetic deshielding at H2 in **1′** in this particular coupling (Fig. 2). Notably, this coupling strongly resembles the dispersive side-on Au3···H2–C2 NOCV interaction channel discussed above (Fig. 1d). Overall, HOMO-1 in **1′** is responsible for ~2 ppm of paramagnetic deshielding at H2, while similar Au–P π-back-bonding orbital (HOMO-1) in **2′** contributes only by 0.2 ppm. An analogous mechanism is likely to be responsible also for the deshielding resonance at C2.

**Fig. 2:**
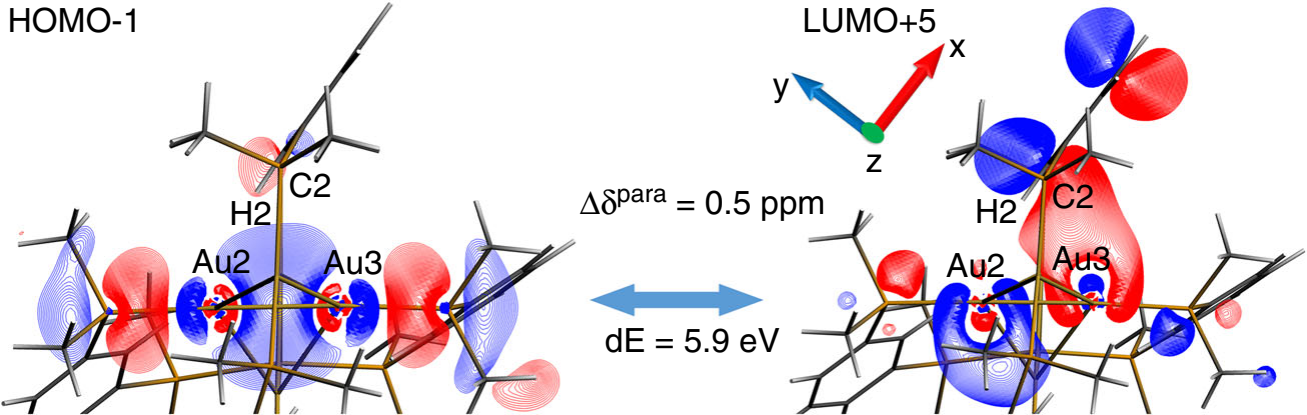
An example of Ramsey-type MO ↔ MO* coupling in **1′**. Orbitals are cut along Au2–H2–Au3 plane

We conclude that the short Au···H contacts in **1** are an example of a weak (~1 kcal/mol per contact) auride-like···hydrogen interaction, with small overall (~4 kcal/mol) stabilizing effect on the cluster structure. Instead, the stabilizing effect can be attributed to the dispersion interactions among the P(Ph)_2_ groups, as documented previously.^10^ Distinct δ(^1^H) NMR deshielding of C–H groups in contact with Au_6_ cluster in **1** as compared with the precursor **2** is due to (a) the differential ED at the H2 atom in **1** as compared to precursor **2**, and (b) side-on orbital interactions between nearby Au3 atom and H–C MOs that increase the efficiency of the local Ramsey-type deshielding paramagnetic couplings in molecule **1** as compared with corresponding couplings in precursor **2**.

## Supplementary information

Supplementary Information

## Data Availability

Supplementary Information: Comparison of fully relaxed structures of **1** and **1′** (Supplementary Fig. 1). Calculated NMR chemical shifts and comparison with experimental data (Supplementary Table 1). Details of atomic charges and charge redistribution between atoms in studied systems (Supplementary Tables 2–4). Frontier orbitals of model system **1′** (Supplementary Fig. 2). All computational data are available from the authors on request.
