# Supplementary material for: 1H NMR is not a proof of hydrogen bonds in transition metal complexes
Source: Nat Commun. 2019 Apr 9;10:1643. doi: 10.1038/s41467-019-09625-9 (PMC6456571; doi:10.1038/s41467-019-09625-9)
Supplement: Supplementary file 1 — Supplementary Information [file 41467_2019_9625_MOESM1_ESM.pdf]

Supplementary Information

^{1}H NMR is Not a Proof of Hydrogen Bonds in Transition Metal Complexes

J. Vícha,[a,*] C. Foroutan-Nejad,[b] M. Straka[c,*]

[a]*Centre of Polymer Systems, University Institute, Tomas Bata University in Zlín, Třída T. Bati, 5678, CZ-76001, Zlín, Czech Republic.*

[b]*Department of Chemistry, Faculty of Science, Masaryk University, Kamenice 5, CZ – 62500 Brno, Czech Republic.*

[c]*Institute of Organic Chemistry and Biochemistry of the Czech Academy of Sciences, Flemingovo nám. 2, CZ-16610, Prague, Czech Republic*

Email: jvicha@utb.cz, straka@uochb.cas.cz

Supplementary Methods

Molecular structures were optimized in Turbomole 7.0[1] using the PBE0 functional[2,3] and def2-TZVPP basis sets[4] with relativistic effective core potential (ECP)[5] for the Au atoms replacing 60 core electrons and the dispersion correction (D3) by Grimme.[6] An implicit conductor-like screening solvent model (COSMO)[7] was used. ETS-NOCV and Mulliken charge analyses were performed using ADF2017 package[8,9] at PBE0/TZP level of theory.

Calculations of NMR chemical shifts were performed in ADF2017 package using PBE40 functional, i.e., the standard PBE0 functional with the exact-exchange admixture set to 40% with SO-ZORA to incorporate relativistic effects.[10–13] This level is referred to as the PBE40/TZP level in the manuscript. Molecular orbital analysis of NMR chemical shifts was performed at the same level, for details see ref. 14.

To analyze the electron density distribution of the model systems, single-point computations were performed at PBE0 and PBE levels combined with def2-TZVPP and def2-SVP basis sets via Gaussian 09.[15] The wavefunction was modified by adding missing core electrons of the gold atoms manually and analyzing the wavefunction by AIMAll[16] package within the context of the quantum theory of atoms in molecules, QTAIM.[17]

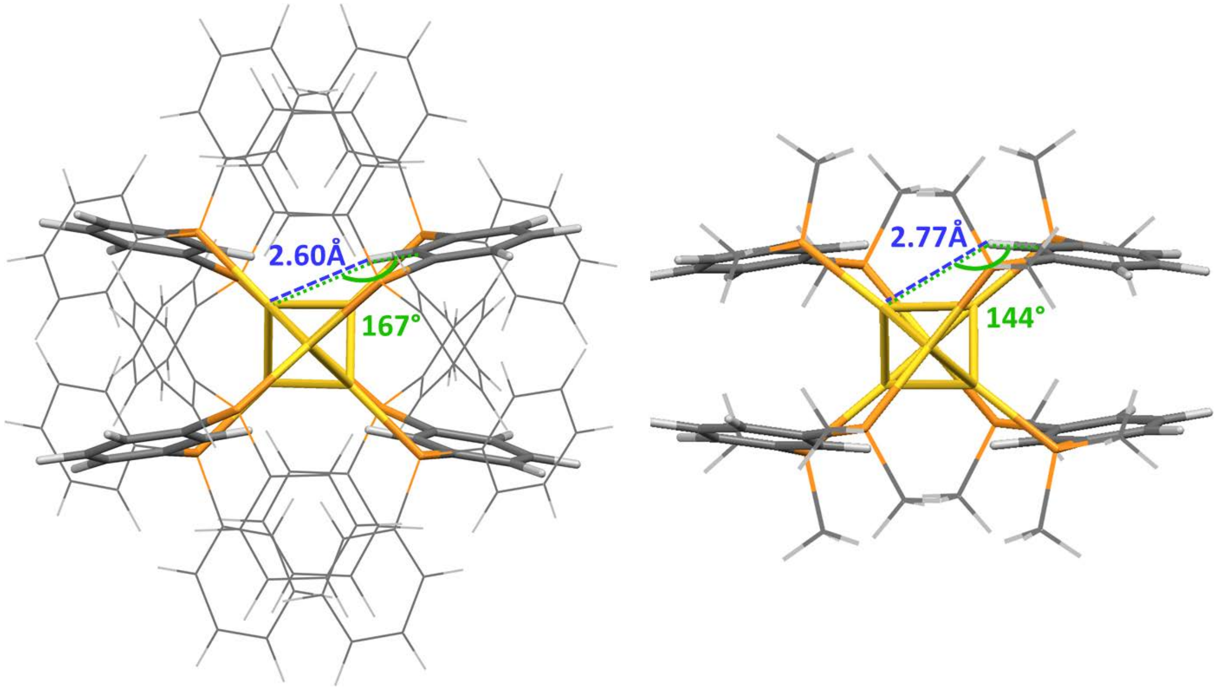

Supplementary Figure 1: Orientation of central phenyl rings relative to central Au_6 cluster in **1** and fully relaxed structure of **1’**, where $P(Ph_2)$ were replaced by $P(Me_2)$. Calculated at PBE0/TZP level.

Supplementary Table 1. Calculated and experimental NMR chemical shifts at H2 and C2 nuclei in **1**, **2**, **1’** and **2’** and their respective differences, $\Delta_{1\text{-}2}$ and $\Delta_{1'\text{-}2'}$ (ppm).

Chem. Shift	**1**	**2**	$\Delta_{1\text{-}2}$	**1’**	**2’**	$\Delta_{1'\text{-}2'}$	**1** (Exp.)	**2** (Exp.)	$\Delta Exp_{1\text{-}2}$
δ (H2)	12.34	7.93	4.41	11.45	7.85	3.60	11.57	7.20	4.37
δ (C2)	151.4	139.6	11.8	147.7	136.2	11.5	147.3	-	-

Supplementary Table 2. Charge transfer between Au2 and its neighboring atoms upon formation of **1**.

Atom/Atom	Au1	Au3	Au4	Au5	P9	H2
Au2	-0.0281	-0.0046	-0.0005	-0.0000	-0.1642	0.0371

Supplementary Table 3. QTAIM atomic charges for **1’** and **2’** and their differences Δ^q calculated at PBE0/def2SVP level.

Atom/System	C1	C2	C3	C4	C5	C6	H2	H4	H5	H6
**1’**	-0.670	-0.011	-0.668	0.015	0.026	0.016	0.042	0.015	0.030	0.013
**2’**	-0.672	0.025	-0.674	0.023	0.034	0.026	0.012	0.015	0.009	0.003
Δ^q	0.002	-0.036	0.006	-0.008	-0.018	-0.010	0.030	0.00	0.021	-0.010
Atom/System	P1	P2	Au1	Au2	Au3	Au4	Au5	Au6	ΣqAu	
**1’**	2.027	2.026	-0.120	-0.159	-0.159	-0.159	-0.159	-0.120	-0.88	
**2’**	2.130	2.136	0.090	-	-	0.084	-	-		

Δ^M	-0.103	-0.110	-0.210	-	-	-0.243	-	-

Supplementary Table 4. Mulliken charges for **1’** and **2’** and the differences between them, Δ^M, calculated at PBE0/TZP level.

Atom/System	C1	C2	C3	C4	C5	C6	H2	H4	H5	H6
**1’**	-0.171	0.228	-0.195	0.152	0.117	0.148	-0.061	-0.082	-0.096	-0.092
**2’**	-0.145	0.225	-0.170	0.117	0.098	0.129	-0.087	-0.056	-0.096	-0.076
Δ^M	-0.026	0.003	-0.025	0.035	0.019	0.019	0.026	-0.026	0.000	-0.016
Atom/System	P1	P2	Au1	Au2	Au3	Au4	Au5	Au6	ΣqAu	
**1’**	0.446	0.410	0.041	-0.136	-0.119	-0.170	-0.089	-0.035	-0.508	
**2’**	0.374	0.344	0.187	-	-	0.177	-	-		
Δ^M	0.072	0.066	-0.146	-	-	-0.347	-	-		

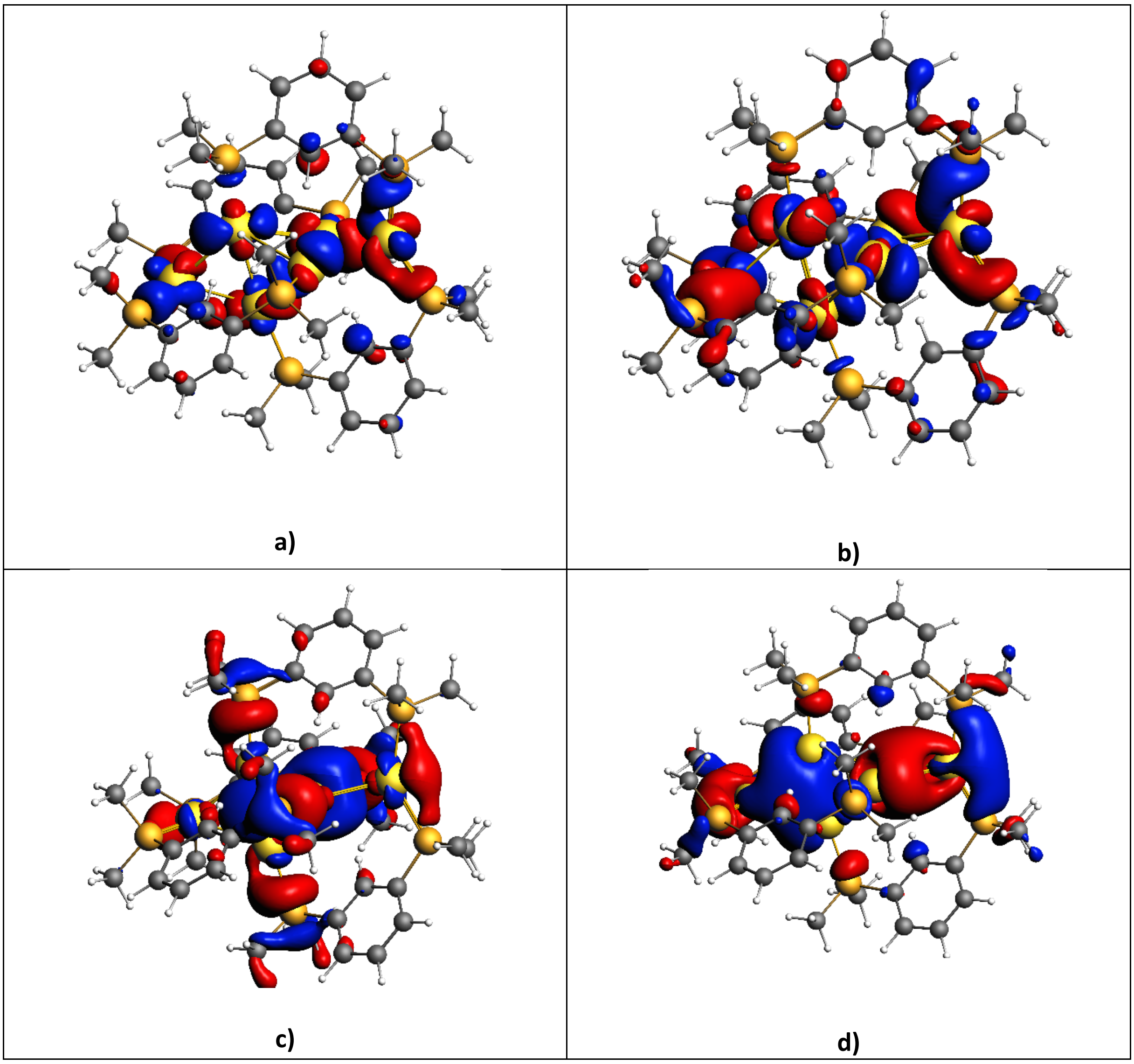

Supplementary Figure 2: a) HOMO-6, b) HOMO-2 (top), c) HOMO-1 and d) HOMO of **1'**.

Supplementary References:

1. Furche, F. *et al.* Turbomole. *Wiley Interdiscip. Rev. Comput. Mol. Sci.* **4,** 91–100 (2014).
2. Adamo, C. & Barone, V. Toward reliable density functional methods without adjustable parameters: The PBE0 model. *J. Chem. Phys.* **110,** 6158–6170 (1999).
3. Adamo, C., Scuseria, G. E. & Barone, V. Accurate excitation energies from time-dependent density functional theory: Assessing the PBE0 model. *J. Chem. Phys.* **111,** 2889–2899 (1999).
4. Weigend, F. & Ahlrichs, R. Balanced basis sets of split valence, triple zeta valence and quadruple zeta valence quality for H to Rn: Design and assessment of accuracy. *Phys. Chem. Chem. Phys.* **7,** 3297–3305 (2005).
5. Andrae, D., Häußermann, U., Dolg, M., Stoll, H. & Preuß, H. Energy-adjustedab initio pseudopotentials for the second and third row transition elements. *Theor. Chim. Acta* **77,** 123–141 (1990).
6. Grimme, S. Semiempirical GGA-type density functional constructed with a long-range dispersion correction. *J. Comput. Chem.* **27,** 1787–1799 (2006).
7. Klamt, A. & Schüürmann, G. COSMO: a new approach to dielectric screening in solvents with explicit expressions for the screening energy and its gradient. *J. Chem. Soc. Perkin Trans. 2* 799–805 (1993).
8. Autschbach, J. & Zheng, S. Analyzing Pt chemical shifts calculated from relativistic density functional theory using localized orbitals: The role of Pt 5d lone pairs. *Magn. Reson. Chem.* **46,** S45–S55 (2008).
9. Autschbach, J. Analyzing NMR shielding tensors calculated with two-component relativistic methods using spin-free localized molecular orbitals. *J. Chem. Phys.* **128,** 164112–164123 (2008).
10. Pawlak, T., Munzarová, M. L., Pazderski, L. & Marek, R. Validation of Relativistic DFT Approaches to the Calculation of NMR Chemical Shifts in Square-Planar Pt2+ and Au3+ Complexes. *J. Chem. Theory Comput.* **7,** 3909–3923 (2011).

11. Vícha, J., Patzschke, M. & Marek, R. A relativistic DFT methodology for calculating the structures and NMR chemical shifts of octahedral platinum and iridium complexes. *Phys. Chem. Chem. Phys.* **15,** 7740–7754 (2013).
12. Pawlak, T., Niedzielska, D., Vícha, J., Marek, R. & Pazderski, L. Dimeric Pd(II) and Pt(II) chloride organometallics with 2-phenylpyridine and their solvolysis in dimethylsulfoxide. *J. Organomet. Chem.* **759,** 58–66 (2014).
13. Vícha, J. *et al.* Structure, solvent, and relativistic effects on the NMR chemical shifts in square-planar transition-metal complexes: assessment of DFT approaches. *Phys. Chem. Chem. Phys.* **17,** 24944–24955 (2015).
14. Vícha, J., Komorovsky, S., Repisky, M., Marek, R. & Straka, M. Relativistic Spin–Orbit Heavy Atom on the Light Atom NMR Chemical Shifts: General Trends Across the Periodic Table Explained. *J. Chem. Theory Comput.* **14,** 3025–3039 (2018).
15. Frisch, M. J. *et al. Gaussian 09*. (Gaussian Inc. Wallingford CT 2009).
16. *Keith, T. A. AIMAll, Gristmill Software, Overland Park KS, USA, 2017 (aim.tkgristmill.com)*.
17. Bader, R. F. W. *Atoms in Molecules: A Quantum Theory*. (Clarendon Press, Oxford; New York, 1990).
